# Population health needs as predictors of variations in NHS practice payments: a cross-sectional study of English general practices in 2013–2014 and 2014–2015

**DOI:** 10.3399/bjgp16X688345

**Published:** 2016-11-22

**Authors:** Louis S Levene, Richard Baker, Andrew Wilson, Nicola Walker, Kambiz Boomla, M John G Bankart

**Affiliations:** Department of Health Sciences, College of Medicine, Biological Sciences and Psychology, University of Leicester, Centre for Medicine, Leicester.; Department of Health Sciences, College of Medicine, Biological Sciences and Psychology, University of Leicester, Centre for Medicine, Leicester.; Department of Health Sciences, College of Medicine, Biological Sciences and Psychology, University of Leicester, Centre for Medicine, Leicester.; Department of Health Sciences, College of Medicine, Biological Sciences and Psychology, University of Leicester, Centre for Medicine, Leicester.; Clinical Effectiveness Group, Centre for Primary Care and Public Health, Queen Mary University of London, London.; Department of Health Sciences, College of Medicine, Biological Sciences and Psychology, University of Leicester, Centre for Medicine, Leicester.

**Keywords:** funding, health services needs, inequalities, populations, underserved, primary health care, workload

## Abstract

**Background:**

NHS general practice payments in England include pay for performance elements and a weighted component designed to compensate for workload, but without measures of specific deprivation or ethnic groups.

**Aim:**

To determine whether population factors related to health needs predicted variations in NHS payments to individual general practices in England.

**Design and setting:**

Cross-sectional study of all practices in England, in financial years 2013–2014 and 2014–2015.

**Method:**

Descriptive statistics, univariable analyses (examining correlations between payment and predictors), and multivariable analyses (undertaking multivariable linear regressions for each year, with logarithms of payments as the dependent variables, and with population, practice, and performance factors as independent variables) were undertaken.

**Results:**

Several population variables predicted variations in adjusted total payments, but inconsistently. Higher payments were associated with increases in deprivation, patients of older age, African Caribbean ethnic group, and asthma prevalence. Lower payments were associated with an increase in smoking prevalence. Long-term health conditions, South Asian ethnic group, and diabetes prevalence were not predictive. The adjusted *R*^2^ values were 0.359 (2013–2014) and 0.374 (2014–2015). A slightly different set of variables predicted variations in the payment component designed to compensate for workload. Lower payments were associated with increases in deprivation, patients of older age, and diabetes prevalence. Smoking prevalence was not predictive. There was a geographical differential.

**Conclusion:**

Population factors related to health needs were, overall, poor predictors of variations in adjusted total practice payments and in the payment component designed to compensate for workload. Revising the weighting formula and extending weighting to other payment components might better support practices to address these needs.

## INTRODUCTION

The share of total NHS expenditure allocated to general practice was 8% (£8.8 billion) in 2013–2014,^1^ steadily declining from 14% in 2005–2006 and 10.5% in 2010–2011. In 2010–2014, however, total NHS spending increased by 4.4%^1^^–^3 and UK primary care consultation rates increased by 10.5%.^4^

From the total NHS expenditure allocated to general practice, payments are made to each practice. In England, General Medical Services (GMS) practices have a standard, nationally negotiated contract, with some local flexibility to opt in or out of providing certain services. Since 2004 the GMS contract^5^ has included a global sum allocation formula (also known as Carr-Hill), which aims to ensure that funding reflects practices’ workloads and reimburses the *‘... unavoidable costs of delivering … care to the local population’*.^6^ Weighting includes adjustments for age and sex structure, morbidity and mortality measures, and list turnover. Ethnic group and deprivation measures are not included, as reliable data on the workload implications were not available in 2004, although previous contracts included area-based weighting for workload associated with deprivation, as well as for age and sex.^7^^,^^8^ To protect practices from income loss from the contract, a ‘minimum practice income guarantee’ (MPIG) was included.

The 2004 contract also included a substantial pay-for-performance element. The Quality and Outcomes Framework (QOF) rewards practices for the provision of ‘quality care’.^5^ Although participation is voluntary, most practices take part in QOF. Practices can also opt in to provide a range of Enhanced Services (ES), intended to reduce the burden on secondary care. ES include Directed (nationally determined) and Local (commissioned locally and which vary between areas) Services.^9^ Both QOF and ES have been updated regularly since 2004.

The Personal Medical Services (PMS) contract was introduced in 1998 as a local alternative to the GMS contract. PMS contracts are voluntary, locally negotiated contracts between practices and primary care administrative organisations, allowing flexible service provision in accordance with specific local circumstances.^10^ The Alternative Provider Medical Services (APMS) contract is a more flexible contract, open to a wider range of providers including the independent sector.^11^ Neither PMS nor APMS practices receive the GMS global sum.

Health needs are *‘... objectively determined deficiencies in health that require health care, from promotion to palliation’*.^12^ These needs are linked to adverse health outcomes, strongly predicted by socioeconomic deprivation,^13^ which are associated with earlier and greater multimorbidity,^14^ including an up to 18-year gap in disability-free life expectancy between most and least deprived populations.^15^ Health inequalities persist, despite absolute and relative decreases in all-cause mortality in lower socioeconomic groups between 1990 and 2010.^16^ Adverse health outcomes are more likely in non-white British ethnic groups, with increasing deprivation and age as important determinants, but patterns vary between health conditions and by sex within individual ethnic groups.^17^

**How this fits in**

Funding allocation to English general practices uses a formula designed to compensate for workload, but without measures of socioeconomic deprivation and ethnic group. This study shows that population factors related to health needs were poor predictors, overall, of variations in practice payments. The directions of the predictive effects of such population factors were inconsistent with each other. Revising the formula could help practices to deal with population health needs and reduce health inequalities.

NHS primary care is currently under mounting professional and financial pressures. In April 2016 NHS England announced a 5-year plan to increase investment in general practice.^18^ If practices are to help in reducing health inequalities, then allocation of new and existing funding should take account of population needs.

The present study aimed to determine whether variations in total NHS payments to English general practices in 2013–2015 were predicted by factors related to the health needs of populations. In addition, predictions by these factors of variations in total payments were compared with variations in GMS global sum payments, the component designed to compensate for workload. The study was exploratory because many variables were examined, and, thus, multiple hypotheses were tested.

## METHOD

### Overview

A cross-sectional study was undertaken across all practices in England, repeated for two consecutive financial years, 2013–2014 and 2014–2015.

### Dependent variables

The main dependent variable in each year was the adjusted total payment per registered patient, calculated from the sum of all payments due to a practice for providing NHS services (using data from the Health and Social Care Information Centre [HSCIC] payments system), then subtracting pensions, levies, prescription charge income, and premises payments; finally, this remainder was divided by the number of registered patients.^19^^,^^20^

The additional analyses were limited to practices with a GMS contract, as only these practices were paid the global sum. The dependent variable was the global sum (plus MPIG) per registered patient. Also, prediction of variations in adjusted total payments per patient in GMS practices was compared with prediction of variations in all practices.

### Independent variables

Variable selection was determined by data availability and relevance to the research questions, using a model in which population factors strongly predict health outcomes.^26^ The data sources of variables are given in Table 1. The population variables covered:

- socioeconomic factors: deprivation, using the English Index of Multiple Deprivation (IMD),^21^ unemployment,^22^ long-term sick or claiming disability,^22^ self-reported confidence in managing health;^22^
- demographic factors: age ≥75 years,^21^ ethnic group (African Caribbean; South Asian);^22^
- lifestyle factor: percentage of smokers; and^22^
- morbidity factors: self-reported with longstanding health condition,^22^ and prevalences from QOF registers of diabetes, heart failure, asthma, and chronic obstructive pulmonary disease (COPD).^23^^,^^24^

**Table 1.** Descriptive statistics of dependent and independent variables used in analyses^a^

**Variable**	**Data source**	**Practices with data, *n***	**Mean**	**SD**	**Median**	**IQR**
**Dependent**						
Adjusted total payments per patient (all contracts) (datasheet included practices with payments for both years)	HSCIC^19^^,^^20^	7693			£102.64 £105.79	£92.27–118.05 £96.35–121.38

Global sum plus MPIG payments per patient (GMS contracts)	HSCIC^19^^,^^20^	4451 4451			£67.53 £74.67	£63.77–72.02 £70.48–79.48

Adjusted total payments per patient (GMS contracts only)	HSCIC^19^^,^^20^	4451 4451			£98.14 £102.61	£89.25–113.59 £93.69–116.01

**Population**						
IMD 2010 score (used for 2013–2014)^b^ IMD 2015 score (used for 2014–2015)^b^	Public Health England.^21^ Individual practices’ scores were calculated by the Office for National Statistics, using patients’ postcodes	7575 7584			21.64 21.74	13.60–31.83 13.88–31.51

Percentage of practice register aged ≥75 years	Public Health England^21^	7585 7584	7.61 7.66	3.12 3.17		

Percentage African Caribbean	General Practice Patient Survey reports (weighted) for both years^22^	7513 7430			0.86 0.84	0.00–3.85 0.00–3.99

Percentage South Asian	General Practice Patient Survey^22^	7485 7513			2.20 1.39	0.00–7.80 0.00–5.58

Percentage of smokers	General Practice Patient Survey^22^	7678 7668	17.79 17.01	6.84 6.87		

Percentage with self-reported long-term condition	General Practice Patient Survey^22^	7689 7684	53.96 54.01	7.77 8.00		

Percentage self-reported confident about managing own health	General Practice Patient Survey^22^	7689 7684			92.91 93.02	89.85–95.21 89.81–95.39

Percentage self-reported unemployed	General Practice Patient Survey^22^	7675 7667			4.9 4.3	2.28–8.95 1.86–8.01

Percentage self-reported on long-term sick or disability register	General Practice Patient Survey^22^	7639 7628			4.02 3.88	2.22–6.47 2.12–6.37

Percentage on practice diabetes register	HSCIC–QOF annual reports^23^^,^^24^	7573 7586	6.47 6.65	1.85 1.91		

Percentage on practice CHD register^c^	HSCIC–QOF annual reports^23^^,^^24^	7573 7585	3.32 3.27	1.14 1.13		

Percentage on practice CVA register^c^	HSCIC–QOF annual reports^23^^,^^24^	7573 7585	1.69 1.70	0.64 0.65		

Percentage on practice heart failure register	HSCIC–QOF annual reports^23^^,^^24^	7573 7581			0.68 0.69	0.49–0.89 0.50–0.90

Percentage on practice asthma register	HSCIC–QOF annual reports^23^^,^^24^	7573 7587	5.89 5.95	1.29 1.32		

Percentage on practice COPD register	HSCIC–QOF annual reports^23^^,^^24^	7573 7581			1.72 1.75	1.20–2.33 1.23–2.39

**Practice**						
Number of registered patients	HSCIC^19^^,^^20^	7693 7693			6342 6441	3819–9687 3891–9776

WTE GPs per 10 000 patients — 2014 only (calculated by dividing WTE number in practices by number of registered patients, then multiplying by 10 000)	NHS workforce statistics^25^	7649			5.99	4.73–7.43

WTE nurses per 10 000 patients — 2014 only (calculated as above)	NHS workforce statistics^25^	7125			2.42	1.78–3.17

WTE staff per 10 000 patients — 2014 only (calculated as above)	NHS workforce statistics^25^	7125			15.24	12.80–18.15

Contract type – GMS^d^	HSCIC^19^^,^^20^	4405				
Contract type – PMS^d^	HSCIC^19^^,^^20^	3085				
Contract type – APMS^d^	HSCIC^19^^,^^20^	203				

Geographical subregion – north^d,e^	HSCIC^19^^,^^20^	2310				
Geographical subregion – Midlands^d,e^	HSCIC^19^^,^^20^	2254				
Geographical subregion – south^d,e^	HSCIC^19^^,^^20^	3129				

**Performance**						
Total QOF points achieved^f^	HSCIC–QOF annual reports^23^^,^^24^	7573			866.67	828.28–887.15
900 points maximum in 2013–2014		7586			542.83	523.41–553.44
559 points maximum in 2014–2015						

Percentage on practice hypertension register	HSCIC–QOF annual reports^23^^,^^24^	7573 7587	13.96 14.02	3.58 3.59		

Percentage of hypertension register with last blood pressure reading ≤150/90 mmHg	HSCIC–QOF annual reports^23^^,^^24^	7676 7587			80.50 81.06	75.68–84.28 77.35–84.37

Percentage of diabetes register with last HbA1c ≤7.5% (59 mmol/mol)	HSCIC–QOF annual reports^23^^,^^24^	7676 7586			61.84 70.41	56.72–66.45 63.64–76.41

Percentage unable to obtain appointment	General Practice Patient Survey^22^	7683 7672			9.53 9.53	5.71–4.11 5.66–14.70

Percentage able to see a GP or nurse within 48 hours	General Practice Patient Survey^22^	7583 7584	51.01 48.68	14.95 14.93		

Percentage having a preferred GP	General Practice Patient Survey^22^	7688 7683	53.32 50.47	12.89 13.16		

Percentage reporting good appointment ‘experience’	General Practice Patient Survey^22^	7583 7584	76.33 75.01	12.83 13.47		

a In each cell, values in the first line are for 2013–2014 and in the second line for 2014–2015. For the continuous variables, if the distribution was skewed (not normal) on visual inspection of the histogram, then the median and interquartile range are presented rather than the mean and standard deviation.

b Index of Multiple Deprivation (IMD) is the official measure of relative deprivation for small areas. IMD ranks each small area (called Lower-layer Super Output Areas [LSOA] of which there are 32 844 with an average of 1500 residents each) in England. IMD combines information from seven domains: income deprivation; employment deprivation; education, skills, and training deprivation; health deprivation and disability; crime; barriers to housing and services; and living environment deprivation.

c Dropped from the final model.

d As these variables are nominal, only numbers of practices are provided.

e There are 13 geographical subregions in England, which were collapsed into three larger regional identities: north, Midlands, and south. North included four subregions: Cheshire and Merseyside, Cumbria and north east, Lancashire and Greater Manchester, and Yorkshire and Humber. Midlands included four subregions: central Midlands, east Midlands, west Midlands, and north Midlands. South included five subregions: London, Wessex, south central, south east, and south west.

f As part of the 2014–2015 GMS contract changes, a total of 40 QOF indicators were retired, ‘releasing’ 341 points. The resource from these points was transferred to the global sum and to ES, including a new ES aimed at avoiding unplanned admissions and delivering proactive case management for vulnerable people. APMS = Alternative Provider Medical Services. CHD = coronary heart disease. COPD = chronic obstructive pulmonary disease. CVA = cerebrovascular accident. ES = Enhanced Services. GMS = General Medical Services. HSCIC = Health and Social Care Information Centre. IQR = interquartile range. MPIG = minimum practice income guarantee. PMS = Personal Medical Services. QOF = Quality and Outcomes Framework. SD = standard deviation. WTE = whole-time equivalent.

To explain as much as possible of the variation in payments, two further groups of variables were added comprising practice^19^^,^^20^^,^^25^ and performance factors.^22^^,^^23^ Descriptions and data sources for these variables are given in Table 1. QOF hypertension registers were used as performance variables because hypertension is under-detected. QOF hypertension register prevalence was 13.7% in 2013–2014^23^ compared with a Health Survey for England estimated hypertension prevalence of 28.6% in 2013;^27^ thus, these registers measure performance in detection more than true prevalence. Two of the practice factors were nominal variables: contract type and geographical subregion.^19^^,^^20^ The remaining variables were treated as continuous in the statistical models.

Descriptive statistics, univariable analyses (examining correlations between payment and each predictor, and between IMD scores and payments), and multivariable analyses (undertaking a multivariable linear regression for each year) were performed. Performance factors were excluded when the dependent variable was the global sum, as this is not based on performance. The assumptions of linearity, normality of residuals, and homogeneity of variance were checked. Multicollinearity was checked, and variables with a 1/Variance Inflation Factor (that is, tolerance) value of <0.2 were omitted from the final model. STATA 14 statistical software was used.

## RESULTS

### Descriptive statistics

Payment data were published in England for 8060 practices in 2013–2014 and 7959 practices in 2014–2015. A small number received implausibly large or small (including negative) payments per patient, and were treated as erroneous and omitted. On this basis, practices with fewer than 500 patients and practices with total adjusted payments per patient of <£10 or >£500 in either year were excluded, leaving 7693 practices for analysis in the 2 years. Further practices were excluded because of missing data, with numbers per analysis provided in the tables.

The distribution of adjusted total payments per patient was slightly positively skewed in both years. Although these payments increased between 2013–2014 and 2014–2015, the percentage paid for the performance-related components declined from 21.9% to 19.1%: the QOF-derived percentage declined from 12.9% to 9.0%, whereas the ES-derived percentage increased from 8.1% to 10.0%. The numbers of practices with GMS, PMS, or APMS contracts were 4440, 3126, and 221, respectively. The number of practices in the north of England, Midlands, and south of England were 2337, 2299, and 3151, respectively. Table 1 summarises the descriptive statistics for all the variables.

### Univariable analyses

The Spearman correlation coefficient between payments in the 2 years was strong (ρ 0.86, *P*<0.01) (Table 2). The correlations between total payments and the independent variables were mostly small, but significant because of the large sample size.

**Table 2.** Spearman correlation coefficients (*P*-value) between payments and IMD score

**Financial year**	**Adjusted total payments**	**QOF payments**	**ES payments**	**Global sum + MPIG payments (GMS practices only)**
2013–2014	0.0003 (0.98)	0.0006 (0.96)	−0.15 (*P*<0.01)	0.27 (*P*<0.01)
2014–2015	0.023 (0.045)	0.0066 (0.57)	−0.18 (*P*<0.01)	0.30 (*P*<0.01)

ES = Enhanced Services. GMS = General Medical Services. IMD = Index of Multiple Deprivation. MPIG = minimum practice income guarantee. QOF = Quality and Outcomes Framework.

**Figure 1.**
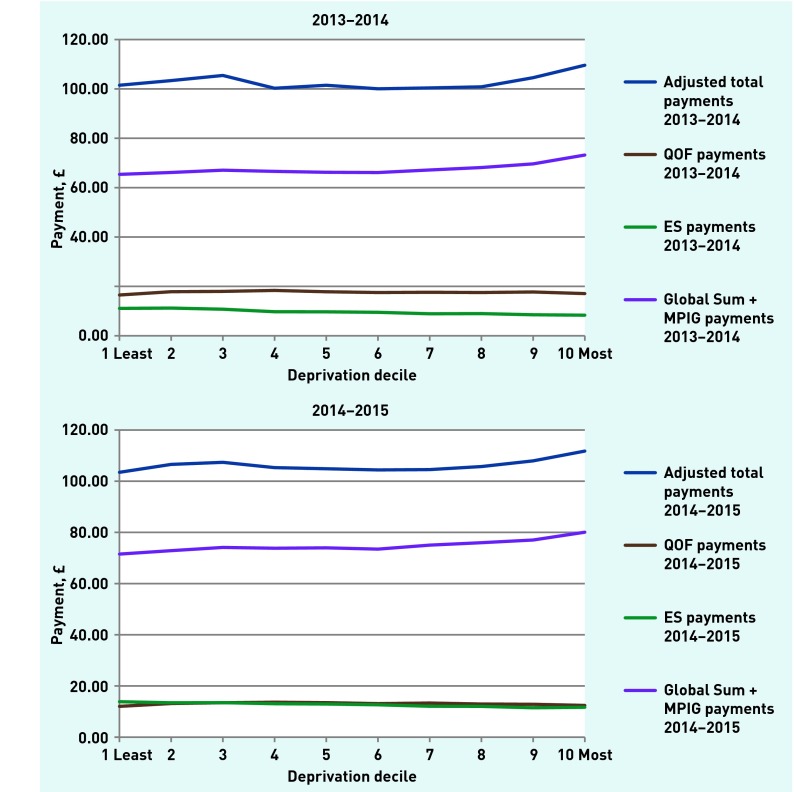
***Medians of payment types by ascending IMD decile. ES = Enhanced Services. IMD = Index of Multiple Deprivation. MPIG = minimum practice income guarantee. QOF = Quality and Outcomes Framework.***

IMD scores were not correlated with total or QOF payments. The Spearman correlation coefficients were negative between IMD scores and ES payments, and positive between IMD scores and global sum payments (all *P*<0.01). When all practices were divided into deciles by IMD scores, however, the trends in the median values of total, global sum, and ES payments showed most change across the two most deprived deciles (Figure 1).

### Multivariable analyses

Natural logarithm transformations were undertaken, as the values of the dependent variables were positively skewed. This transformation generally improved normality. For adjusted total payments in all practices, the adjusted *R*^2^ values were 0.359 (2013–2014) and 0.374 (2014–2015); this explained only just over one-third of payment variations, despite a wide range of plausibly relevant variables being modelled.

Table 3 shows the variables predicting variations in adjusted total payments. Higher payments were associated with increases in deprivation (socioeconomic), patients of older age, African Caribbean ethnic group (demographic), and asthma prevalence (morbidity). Lower payments were associated with increases in smokers (lifestyle) and having a long-term health condition (morbidity). Some population variables were not significant either at all or across both years. These included South Asian ethnic group and the prevalences of diabetes, heart failure, and COPD. QOF coronary heart disease (CHD) and stroke (CVA) prevalences were excluded from the final model, as these variables had high collinearity.

**Table 3.** Linear regression results for 2013–2014 and 2014–2015 (significant independent variables), with dependent variable logarithm of adjusted total payments per registered patient, and including all contract types^a^

**Predictor**	**2013–2014 β coefficient, (95% CI)**	**2014–2015 β coefficient, (95% CI)**
**Population**		
Percentage of list aged ≥75 years	**0.0035 (0.00021 to 0.0067)**	**0.0064 (0.0031 to 0.0095)**
IMD score	**0.00098 (0.00068 to 0.0019)**	**0.00099 (0.00012 to 0.0019)**
Percentage African Caribbean	**0.16 (0.051 to 0.28)**	**0.16 (0.059 to 0.27)**
Percentage of smokers	**−0.36 (−0.47 to −0.26)**	**−0.21 (−0.31 to −0.12)**
Percentage with self-reported long-term condition	**−0.15 (−0.25 to −0.062)**	**−0.11 (−0.19 to −0.022)**
Percentage of self-reported on long-term sick or disability register	−0.21 (−0.44 to 0.0088)	**−0.30 (−0.50 to −0.10)**
Percentage on practice diabetes register	−0.0022 (−0.0079 to 0.0034)	**−0.0069 (−0.012 to −0.0019)**
Percentage on practice asthma register	**0.0073 (0.0021 to 0.012)**	**0.0054 (−0.000063 to 0.010)**

**Practice**		
Number of registered patients	**−2.43e−06 (−3.90e−06 to −9.54e−07)**	**−2.67e−06 (−4.01e−06 to −1.32e−06)**
Contract type: GMS with APMS as reference	**−0.14 (−0.17 to −0.10)**	**−0.18 (−0.21 to −0.14)**
Contract type: PMS with APMS as reference	**−0.089 (−0.13 to −0.052)**	**−0.14 (−0.17 to −0.11)**
Geographical region: Midlands with north as reference	**0.087 (0.072 to 0.10)**	**0.056 (0.042 to 0.07)**
Geographical region: south with north as reference	**0.040 (0.025 to 0.056)**	**0.015 (0.0056 to 0.029)**
WTE GPs per 10 000 patients	**0.016 (0.014 to 0.019)**	**0.015 (0.013 to 0.017)**
WTE staff per 10 000 patients	**0.024 (0.023 to 0.026)**	**0.024 (0.023 to 0.026)**

**Performance**		
Total QOF points achieved	**0.00019 (0.000075 to 0.00030)**	0.00015 (−0.000049 to 0.00034)
Percentage on practice hypertension register	**0.0061 (0.0031 to 0.0092)**	**0.0077 (0.0048 to 0.011)**
Percentage reporting good appointment ‘experience’	**0.0030 (0.0027 to 0.0037)**	**0.0025 (0.0019 to 0.0032)**

Significant values in bold.

a *Adjusted* R*^2^ values: 2013–2014* = *0.359 (6209 practices); 2014–2015* = *0.374 (6282 practices). Mean variance inflation factor: 2.90 in 2013–2014; 2.88 in 2014–2015. Negative figures indicate a reduction in adjusted total payment. APMS = Alternative Provider Medical Services. GMS = General Medical Services. IMD = Index of Multiple Deprivation. QOF = Quality and Outcomes Framework. WTE = whole-time equivalent.*

Higher payments were associated with increases in three practice variables: GP numbers per patient, non-clinical staff numbers per patient, and numbers of registered patients. There was a geographical effect: in 2014–2015, practices in the north were paid 5.8% (8.7% in 2013–2014) and 1.9% (4.0% in 2013–2014) less than practices in the Midlands and in the south, respectively. Higher payments were also associated with increases in two performance variables: hypertension detection and good appointment experience.

Fewer of the variations in global sum payments were accounted for (Table 4). Higher payments were associated with increases in both African Caribbean and South Asian ethnic groups, and numbers of GPs per patient, and non-clinical staff per patient. Lower payments were associated with increases in deprivation, patients of older age, diabetes prevalence, and numbers of registered patients. Smoking prevalence was not predictive.

**Table 4.** Linear regression results for 2013–2014 and 2014–2015, with dependent variable logarithm of the global sum plus MPIG payments per registered patient, and including GMS contract practices only^a^

**Predictor**	**2013–2014 β coefficient (95% CI)**	**2014–2015 β coefficient (95% CI)**
**Population**		
Percentage of list aged ≥75 years	**−0.012 (−0.014 to −0.011)**	**−0.010 (−0.012 to −0.0086)**
IMD score	**−0.0019 (−0.0025 to −0.0014)**	**−0.0011 (−0.0016 to −0.00063)**
Percentage African Caribbean	**0.16 (0.083 to 0.24)**	**0.075 (0.0044 to 0.14)**
Percentage South Asian	**0.15 (0.11 to 0.20)**	**0.089 (0.045 to 0.13)**
Percentage of smokers	0.014 (−0.051 to 0.079)	012 (−0.046 to 0.069)
Percentage with self-reported long-term condition	**−0.092 (−0.15 to −0.037)**	−0.034 (−0.082 to 0.014)
Percentage of self-reported on long-term sick or disability register	−0.080 (−0.22 to 0.058)	**−0.13 (−0.25 to −0.0078)**
Percentage of self-reported confident about managing own health	**−0.11 (−0.22 to −0.0042)**	−0.028 (−0.12 to 0.067)
Percentage of self-reported unemployed	−0.0010 (−0.098 to 0.096)	0.022 (−0.070 to 0.11)
Percentage on practice diabetes register	**−0.0064 (−0.0094 to −0.0034)**	**−0.0041 (−0.0068 to −0.0014)**
Percentage on practice heart failure register	0.0048 (−0.0083 to 0.018)	−0.00039 (−0.012 to 0.011)
Percentage on practice asthma register	0.0016 (−0.0015 to 0.0047)	−0.00014 (−0.0026 to 0.0029)
Percentage on practice COPD register	−0.0041 (−0.0011 to 0.0024)	**−0.0077 (−0.014 to −0.0019)**

**Practice**		
List size	**−3.36e−06 (−4.25e−06 to −2.48e−06)**	**−2.89e−06 (−3.67e−06 to −2.10e−06)**
Geographical region: Midlands with north as reference	**0.016 (0.0065 to 0.025)**	**0.019 (0.011 to 0.027)**
Geographical region: south with north as reference	**0.024 (0.015 to 0.033)**	**0.026 (0.017 to 0.034)**
WTE GPs per 10 000 patients	**0.0060 (0.0047 to 0.0074)**	**0.0050 (0.0038 to 0.0062)**
WTE nurses per 10 000 patients	−0.00047 (−0.0030 to 0.0040)	0.0032 (−5.16e−07 to 0.0065)
WTE staff per 10 000 patients	**0.0043 (0.0035 to 0.0052)**	**0.0037 (0.0029 to 0.0045)**

Significant values in bold.

a *Adjusted* R*^2^ values: 2013–2014* = *0.283 (3530 practices); 2014–2015* = *0.202 (3621 practices). Mean variance inflation factor: 2.06 in 2013–2014, 2.03 in 2014–2015. Negative figures indicate a reduction in payment. COPD = chronic obstructive pulmonary disease. IMD = Index of Multiple Deprivation. MPIG = minimum practice income guarantee. WTE = whole-time equivalent.*

Of variations in adjusted total payments, slightly more were accounted for in GMS practices than in all practices, but not with all the same variables (Table 5). Higher payments were associated with increases in hypertension detection, continuity, and good appointment ‘experience’. Lower payments were associated with an increase in smokers. Age, deprivation, both ethnic group variables, and diabetes prevalence were either not significant at all or only in 1 year.

**Table 5.** Linear regression results for 2013–2014 and 2014–2015 with dependent variable logarithm of adjusted total payments per registered patient, and including GMS contract practices only^a^

**Predictor**	**2013–14 β coefficient (95% CI)**	**2014–15 β coefficient (95% CI)**
**Population**		
Percentage of list aged ≥75 years	−0.00050 (−0.0038 to 0.0046)	**0.0048 (0.00036 to 0.0093)**
IMD score	0.00027 (−0.00098 to 0.0015)	**0.0017 (0.00041 to 0.0030)**
Percentage African Caribbean	0.038 (−0.13 to 0.21)	0.011 (−0.17 to 0.19)
Percentage South Asian	−0.075 (−0.18 to 0.030)	−0.037 (−0.15 to 0.076)
Percentage of smokers	**−0.33 (−0.47 to −0.18)**	**−0.34 (−0.48 to −0.19)**
Percentage with self-reported long term condition	**−0.24 (−0.36 to −0.12)**	−0.047 (−0.17 to 0.078)
Percentage of self-reported on long-term sick or disability register	−0.16 (−0.46 to 0.15)	**−0.48 (−0.79 to −0.17)**
Percentage of self-reported confident about managing own health	0.0046 (−0.24 to 0.25)	0.19 (−0.052 to 0.44)
Percentage of self-reported unemployed	**−0.26 (−0.47 to −0.044)**	−0.24 (−0.47 to 0.0028)
Percentage on practice diabetes register	−0.0045 (−0.0079 to 0.0070)	−0.0022 (−0.0010 to 0056)
Percentage on practice heart failure register	0.022 (−0.0072 to 0.051)	−0.013 (−0.042 to 0.017)
Percentage on practice asthma register	**0.011 (0.0035 to 0.017)**	0.0035 (−0.0037 to 0.011)
Percentage on practice COPD register	−0.0028 (−0.017 to 0.012)	−0.0090 (−0.024 to 0.0062)

**Practice**		
List size	−8.06e−06 (−2.98e−06 to 1.37e−07)	−1.19e−06 (−3.42e−06 to 1.05e−06)
Geographical region: Midlands with north as reference	**0.099 (0.079 to 0.12)**	**0.048 (0.027 to 0.068)**
Geographical region: south with north as reference	**0.062 (0.041 to 0.082)**	0.021 (−0.00016 to 0.043)
WTE GPs per 10 000 patients	**0.020 (0.017 to 0.023)**	**0.017 (0.013 to 0.020)**
WTE nurses per 10 000 patients	**−0.015 (−0.023 to −0.0075)**	**−0.018 (−0.026 to −0.010)**
WTE staff per 10 000 patients	**0.030 (0.028 to 0.032)**	**0.029 (0.026 to 0.031)**

**Performance**		
Total QOF points achieved	0.000064 (−0.000087 to 0.00022)	0.00014 (−0.00014 to 0.00043)
Percentage on practice hypertension register	**0.0090 (0.0050 to 0.013)**	**0.012 (0.0078 to 0.016)**
Percentage of the hypertension register with the last blood pressure reading ≤150/90 mmHg	−0.00031 (−0.0014 to 0.0015)	0.00085 (−0.00071 to 0.0024)
Percentage of the diabetes register with the last HbA1c ≤7.5% (59 mmol/mol)	0.00062 (−0.0011 to 0.00010)	−0.00034 (−0.0014 to 0.00071)
Percentage self-reported unable to obtain appointment	−0.068 (−0.27 to 0.13)	−0.089 (−0.28 to 0.11)
Percentage self-reported able to see a GP or nurse within 48 hours	0.00015 (−0.00036 to 0.00065)	0.00032 (−0.00022 to 0.00085)
Percentage having a preferred GP	**0.091 (0.031 to 0.15)**	**0.12 (0.060 to 0.18)**
Percentage reporting good appointment ‘experience’	**0.0034 (0.0024 to 0.0044)**	**0.0025 (0.0015 to 0.0034)**

Significant values in bold.

a *Adjusted R^2^ values: 2013–2014* = *0.440 (3596 practices); 2014–2015* = *0.392 (3621 practices). Mean variance inflation factor: 2.17 in 2013–2014, 2.20 in 2014–2015. Negative figures indicate a reduction in payment. COPD = chronic obstructive pulmonary disease. IMD = Index of Multiple Deprivation. QOF = Quality and Outcomes Framework. WTE = whole-time equivalent.*

Table 6 shows the independent effects of changes to individual significant variables on income in a hypothetical practice of 7000 patients.

**Table 6.** Effect size: the independent effects of changes to values of significant predictors on total payments in a hypothetical practice of 7000 patients^a^

**Predictor**	**Predicted change in total payment based on a value at quartile 1 of the predictor’s distribution, assuming all other predictors remained at their median**	**Predicted change in total payment based on a value at quartile 3 of the predictor’s distribution, assuming all other predictors remained at their median**
Age ≥75 years	£–5491.21 (−0.76%)	£4811.95 (0.67%)
	£–10 812.45 (−1.46%)	£9458.35 (1.28%)

IMD score	£–5682.24 (−0.79%)	£7209.35 (1.00%)
	£–6173.92 (−0.83%)	£7677.80 (1.04%)

Smokers	£11 070.20 (1.54%)	£–13 118.16 (−1.83%)
	£6452.63 (0.87%)	£–7878.13 (−1.06%)

Black ethnic group	£–1016.04 (−0.14%)	£3538.72 (0.49%)
	£–1025.27 (−0.14%)	£3852.10 (0.52%)

With self-reported long-term condition	£5688.65 (0.79%)	£–5688.65 (−0.79%)
	£4189.69 (0.57%)	£–4056.20 (−0.55%)

On long-term sick or disability register	Not a predictor in 2013–2014	Not a predictor in 2013–2014
	£3932.06 (0.53%)	£–5569.10 (−0.75%)

On diabetes register	Not a predictor in 2013–2014	Not a predictor in 2013–2014
	£50.61 (0.0068%)	£–52.18 (−0.0070%)

On asthma register	£–44.34 (−0.0062%)	£40.69 (0.0057%)
	£–33.7 (−0.0045%)	£32.57 (0.0044%)

List size	£4418.45 (0.61%)	£–5863.86 (−0.82)
	£5059.10 (0.68%)	£–6623.46 (−0.89%)

WTE GPs per 10 000 patients	£–15 008.89 (−2.09%)	£17 178.46 (2.39%)
	£–14 034.61 (−1.90%)	£16 061.15 (2.17%)

WTE staff per 10 000 patients	£–43 943.07 (−6.12%)	£52 711.27 (7.34%)
	£–45 428.78 (−6.13%)	£54 494.37 (7.36%)

Total QOF points achieved	£–4229.76 (−0.59%)	2253.37 (0.31%)
	Not a predictor in 2014–2015	Not a predictor in 2014–2015

On hypertension register	£–94.74 (−0.013)	£88.45 (0.012%)
	£–128.23 (−0.017%)	£123.06 (0.017%)

Reporting good appointment experience	£–208.46 (−0.029%)	£171.78 (0.024%)
	£–176.01 (−0.024%)	£164.34 (0.022%)

a Median total (predicted) payments for a 7000 patient list were £718 480 in 2013–2014 and £740 530 in 2014–2015. In each cell, values in the first line are for 2013–2014 and in the second line for 2014–2015. Numbers inside the parentheses are the percentage changes to total payments. Calculations assumed a reference point of the total payments per patient being at the median (£102.64 in 2013–2014 and £105.79 in 2014–2015).

Equations used in the calculations:^28^

*1. Change in payment (amounts)* = *list size* × *median total payment per patient* × *[e* ^*(β coefficient* × *difference between median and quartile values)*^ −*1].*

*2. Change in payments (percentage)* = *[e* ^*(β coefficient* × *difference between median and quartile values)*^−*1]* × *100 e* = *exponential function (2.71828).*

IMD = Index of Multiple Deprivation. QOF = Quality and Outcomes Framework. WTE = whole-time equivalent.

Residuals from all models were approximately normally distributed, and plots of the residuals versus predicted values showed no pattern.

## DISCUSSION

### Summary

Ideally, funding allocation should help practices to respond better to their entire populations’ health needs. The present multivariable analyses found, however, that population factors related to health needs were poor predictors, overall, of variations in total payments, including English practices with all types of contract, and, in global sum payments, designed to compensate for workload in GMS practices only.

Although deprivation was a predictor in most of the analyses, some other population variables were either not significant or were associated with lower payments when the value of the variable increased. These suggest only a weak association between population factors and funding. Fewer of the variations in global sum payments than in total payments were accounted for, with different significant variables and with increases in some variables (including deprivation) associated with lower payments. Age–sex structure is a substantial element in Carr-Hill, but an increase in patients of older age was associated with lower global sum payments. A geographical differential was also found. Some effect sizes were small, but combinations of several changes could substantially alter funding when applied to average-sized practices.

Deprivation and ethnic group may be correlated with elements in Carr-Hill. Univariable analyses examining deprivation deciles suggest, however, that the weighting helped practices mainly in very deprived areas. Practices serving moderately deprived, largely South Asian populations would receive equivalent total and global sum payments to those serving affluent white populations. Practices in more deprived areas may be less able or willing to undertake ES, and thus not generate additional income by delivering more services.

Practice workloads, strongly driven by demand, are focused mainly on known morbidity and not necessarily on the health needs of whole populations. If weighting of payments aimed primarily at compensating for workload does not include measures related to population health needs, then practices serving populations with greater needs may not receive sufficient funding to tackle these needs and potentially reduce health inequalities.

### Strengths and limitations

This study covered a whole nation, using recent time-matched variables in a model that focused on a specific research question. Comparison of covariates between excluded (because of erroneous or missing data) and included practices showed no major differences.

Only one-third of the variation in total payments was accounted for, despite using a wide range of predictors. Factors explaining the remaining two-thirds of the variation were not identified or measured, because of unknown or unmeasured factors, or the complexity of the formula, or both.

As validated measures of multimorbidity were unavailable, single disease morbidity registers were used, which had limited predictive effect. Adding a multimorbidity variable may weaken the predictive effect of deprivation because of intercorrelation, as deprivation is associated with multimorbidity.^29^ Unmet health needs include deficiencies in identifying morbidity, in accessing health care, and in delivering effective interventions. The lack of multimorbidity measures in populations and in practices limited identification of associations between funding variations and unmet needs, but the present analyses included access and treatment effectiveness measures.

The GP Patient Survey has low response rates. Its methodology has been modified, however, with *‘proportionately stratified, unclustered samples drawn from each practice’*,^30^ and data weighted to account for unequal probability of selection, differences between responders and non-responders, and the demographic characteristics of the eligible population.

### Comparison with existing literature

Higher payments are associated with several indicators of better-quality general practice: lower secondary care usage, higher patient satisfaction,^31^ better Care Quality Commission practice ratings,^32^ and increased numbers of GPs per patient (associated with lower mortality)^26^ and of non-clinical staff per patient. In 2004–2014, the gap in GP numbers per patient narrowed between areas with high and low deprivation, using lower-layer super output areas (LSOA) as the population unit;^33^ the present study was cross-sectional and used practices, a larger population unit not always configured geographically.

Deprivation increases workload: consultation rates of patients aged 50 years in the most deprived quintile equalled those aged 70 years in the least deprived quintile. If weighting the age–sex workload in Carr-Hill included consultation rates by deprivation decile, it could deliver one-third more global sum funding to Tower Hamlets, a deprived borough.^34^

In Scotland there are associations between deprivation and multimorbidity, between deprivation and consultation rates, but not between deprivation and practice funding.^29^ The differences in total payment per patient between the first and third most deprived deciles are similar to the present findings, despite differences between definitions (and use of means) of total payments and in how Scottish IMD is calculated.^29^

### Implications for research and practice

Better measures of population health needs are required. The present findings are important for discussions about allocating additional primary care investment. If public health policies involving primary care are to better address local health needs and succeed in reducing health inequalities, then the following should be considered:

- better alignment of Carr-Hill’s weighting to population health needs, by including, for example, suitable measures of deprivation, population multimorbidity, and ethnic group; and
- extend weighting to other payment components, for example, QOF.

The effects of such changes on health inequalities must be monitored.
